# Association of SGLT2 inhibition with psychiatric disorders: A Mendelian randomization study

**DOI:** 10.1515/med-2025-1278

**Published:** 2025-10-25

**Authors:** Le Liu, Chen Li, Shuang Li, Junkun Zhan, Youshuo Liu

**Affiliations:** Department of Geriatrics, The Second Xiangya Hospital, Central South University, Changsha, Hunan, China; Institute of Aging and Age-related Disease Research, Central South University, Changsha, Hunan, China; Department of Geriatrics, The Second Xiangya Hospital, Central South University, 139 Renmin Middle Road, Furong District, Changsha, Hunan, China

**Keywords:** SGLT2 inhibitor, psychiatric disorder, depression, anxiety disorder, obsessive-compulsive disorder

## Abstract

**Background:**

Some observational studies have reported that sodium–glucose cotransporter 2 (SGLT2) inhibitors may have an impact on psychiatric disorders. This Mendelian randomization (MR) study aims to explore the causal relationship between SGLT2 inhibition and five types of psychiatric disorders.

**Methods:**

Genetic variants associated with the SLC5A2 gene and glycated hemoglobin were selected from the eQTLGen Consortium and Genotype-Tissue Expression datasets. Type 2 diabetes served as a positive control in the application of MR and colocalization analyses to investigate potential causal relationships between SGLT2 inhibition and depression, anxiety disorder, schizophrenia, obsessive-compulsive disorder, and bipolar affective disorder. The impact of glycated hemoglobin on psychiatric disorders was additionally analyzed.

**Results:**

SGLT2 inhibition was associated with an increased risk of anxiety disorder, obsessive-compulsive disorder, and bipolar affective disorder. The effect of SGLT2 inhibition on depression did not reach Bonferroni-corrected significance levels. No association was found between SGLT2 inhibition and schizophrenia.

**Conclusions:**

This study provides genetic evidence supporting that SGLT2 inhibitors increase the risk of obsessive-compulsive disorder, anxiety disorder, and bipolar affective disorder.

## 1 Introduction

Due to the long-term physical and psychological burden caused by illness, patients with chronic diseases are often prone to mental illnesses such as depression and anxiety, which seriously reduces their quality of life and affects the treatment outcomes of their underlying diseases. The mental state of diabetic patients has always been a significant concern for medical professionals. According to reports, diabetes is associated with various mental disorders such as depression, anxiety, and schizophrenia [1–4]. Therefore, the impact of anti-diabetic drugs on psychiatric disorders deserves investigation.

In recent years, sodium–glucose cotransporter 2 (SGLT2) inhibitors, a type of hypoglycemic agents, have become a hot topic in the medical field due to their safe and excellent hypoglycemic effect as well as their protective effects on the hearts and kidneys [5–8]. Common SGLT2 inhibitors include empagliflozin, dapagliflozin, and canagliflozin, which can promote urinary glucose excretion by inhibiting the reabsorption of glucose by the renal proximal tubules, thus reducing blood sugar levels. Some studies have explored the effects of SGLT2 inhibitors on psychiatric disorders. For example, observational studies have shown that the use of SGLT2 inhibitors is associated with a reduced risk of depression in patients with type 2 diabetes mellitus [9,10]. Animal experiment has shown the regulatory effect of SGLT2 inhibitors on depression in rats [11]. However, these studies generally have problems such as the interference of various confounding factors and biases, as well as small sample sizes, resulting in limited evidence quality. Therefore, more effective methods are needed to assess the impact of SGLT2 inhibitors on psychiatric disorders.

Mendelian randomization (MR) is a method for causal inference of genetic variations, which is unlikely to be affected by confounding factors or reverse causality [12]. The randomness of allele assignment makes MR studies similar to randomized controlled trials [13]. Currently, MR studies have been widely used in the fields of endocrinology [14], cardiovascular diseases [15], oncology [16], and others, effectively predicting the relationship between drugs and diseases to guide the clinical application of drugs. Therefore, a MR study was conducted to clarify the effect of SGLT2 inhibitors on multiple psychiatric disorders.

## 2 Methods

### 2.1 Study design

**Figure 1:**
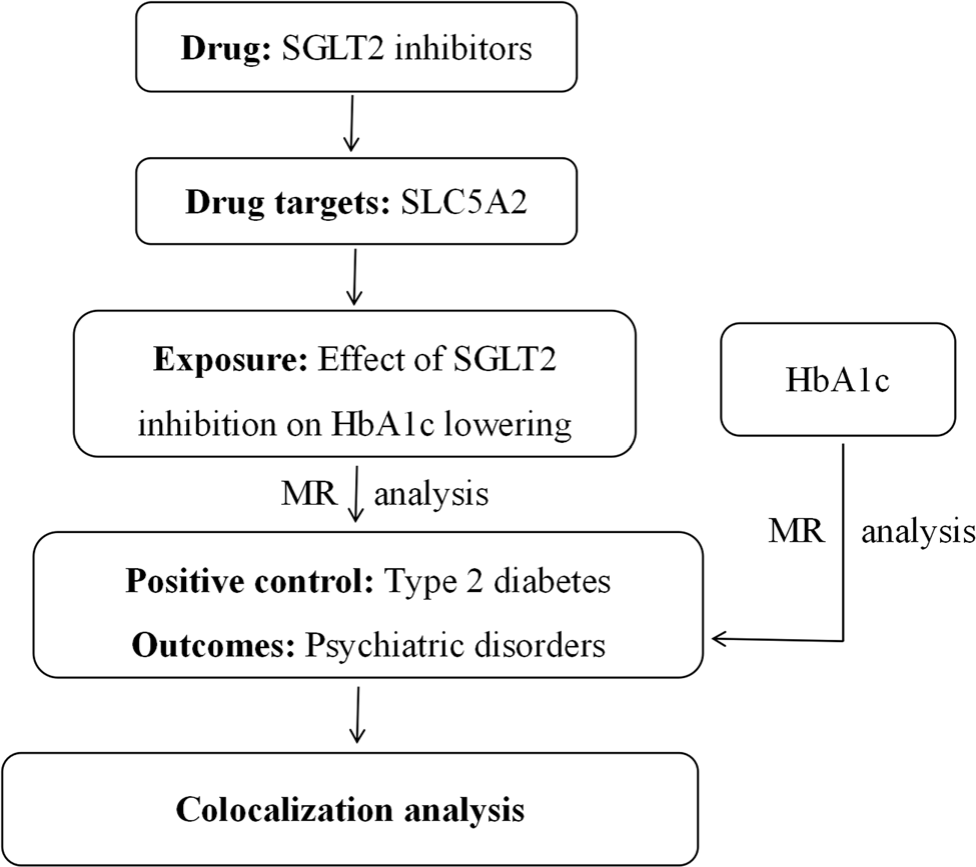
The flowchart of mendelian randomization study design. HbA1c, glycated hemoglobin; SGLT2, sodium–glucose cotransporter 2; MR, mendelian randomization.

The design of this study is divided into the following sections: (1) selection of genetic variants of SGLT2 inhibition; (2) positive validation of genetic variants related to SGLT2 inhibition; (3) conducting MR and colocalization analysis to clarify the causal relationship between SGLT2 inhibition and five types of psychiatric disorders, including depression, anxiety disorder, schizophrenia, obsessive-compulsive disorder, and bipolar affective disorder; and (4) clarifying the impact of glycated hemoglobin (HbA1c) on psychiatric disorders. To ensure the validity of potential causal effects, the conduct of MR analysis must satisfy three fundamental assumptions [17], specifically as follows: (1) the selected genetic variants must be strongly associated with SGLT2 inhibition; (2) the selected genetic variants must be unrelated to confounding factors; and (3) the selected genetic variants should have no direct impact on the risk of psychiatric disorders but only influence the risk through the SLC5A2 drug target. This study followed the Strengthening the Reporting of Observational Studies in Epidemiology Using Mendelian Randomization guidelines [18]. The specific research design is illustrated in Figure 1.

### 2.2 Genetic instruments for SGLT2 inhibition

The screening and identification of SGLT2 inhibition involves the following steps: (1) select genetic variants related to the target gene of SGLT2 inhibitors, SLC5A2 mRNA expression levels, from publicly available data in the eQTLGen Consortium [19] and genotype-tissue expression (GTEx) [20]; (2) evaluate the correlation between the selected SLC5A2 genetic variants and HbA1c, using data from a subgroup of 344,182 unrelated individuals of European descent without diabetes in the UK Biobank (Table S1). Select the SLC5A2 genetic variants that demonstrate significant correlation with HbA1c (*P* < 1 × 10^−4^); (3) after screening and validation, a standard clustering process is conducted to remove variants with very high correlation by using a threshold of correlation between variants <0.3; and (4) the strength of the SLC5A2 genetic variants is estimated using *F*-statistics. An *F*-statistic value of at least 10 suggests the absence of weak instrumental bias.

### 2.3 Instrument validation

To validate the selection of genetic variants, a positive control analysis was conducted using type 2 diabetes as the outcome (as SGLT2 inhibitors are widely used in diabetes treatment). The genome-wide association studies (GWAS) summary data for type 2 diabetes were extracted from the FinnGen database [21], which included 65,085 type 2 diabetes cases and 335,112 controls (Table S1).

### 2.4 Study outcomes

Publicly available case–control summary data for depression (47,696 cases, 359,290 controls), anxiety disorder (44,663 cases, 301,879 controls), schizophrenia (6,708 cases, 398,386 controls), obsessive-compulsive disorder (2,175 cases, 368,054 controls), and bipolar affective disorder (7,569 cases, 359,290 controls) were extracted from the FinnGen database (Table S1) [21]. All GWAS data used in this study were derived from European ancestry.

### 2.5 Statistical analysis

First, the genetic instruments for SGLT2 inhibition were harmonized with the outcome data. Subsequently, analyses were conducted using inverse-variance weighted (IVW), simple mode, weighted mode, and weighted median methods. The IVW method (fixed/random effects) was used as the main analytical tool to evaluate the causal effects of SGLT2 inhibition on the risk of type 2 diabetes and psychiatric disorders [22]. Cochrane’s *Q* statistic for IVW and MR Egger were used to evaluate the heterogeneity of genetic instruments. *P* > 0.05 indicated that there was no significant heterogeneity. The MR Egger method was employed to assess the horizontal pleiotropy of the genetic instruments [23]. *P* > 0.05 indicated that there was no significant horizontal pleiotropy. Additionally, leave-one-out analyses were conducted to evaluate whether individual single-nucleotide polymorphisms (SNPs) would have an impact on the overall estimation of the causal effect.

Bonferroni-corrected significance levels of *P* < 0.01 (0.05/5) were applied to adjust for multiple testing in the analysis of the effects of SGLT2 inhibition on psychiatric disorders. Regarding the psychiatric disorders associated with SGLT2 inhibition discovered in this study, a colocalization analysis was conducted using the coloc R package [24]. This analysis employed a Bayesian model to estimate the posterior probability that SLC5A2 and psychiatric disorders share the same causal variants. Evidence of colocalization was defined as the posterior probability of sharing the same causal variants >50% [16,25].

To determine whether the causal relationship between SGLT2 inhibition and psychiatric disorders is mediated through HbA1c, two-sample MR analysis was performed to assess the association between HbA1c levels and psychiatric disorders. Genetic variants were selected from HbA1c data of 34,182 unrelated European individuals without diabetes from the UK Biobank at a significance level of *P* < 5 × 10^−8^ (Table S1). Subsequently, highly correlated variants were excluded using a linkage disequilibrium threshold of <0.001. The data analysis was conducted using the TwoSampleMR R package (version 0.5.11).

All statistical analyses were conducted using R software (version 4.3.1).

## 3 Results

### 3.1 Instrument validation: Impact of SGLT2 inhibition on type 2 diabetes

In this study, a total of seven independent SNPs were selected as genetic instruments for SGLT2 inhibition (Table S2). After validation, *F*-statistics for each SNP was greater than 14. Through MR analysis, a significant association was observed between SGLT2 inhibition and a reduced risk of type 2 diabetes (*P* = 3.02 × 10^−5^, odds ratio [OR] [95% confidence interval (CI)] = 0.47[0.33, 0.67]) (Table 1). There was no evidence of heterogeneity among the genetic instruments (*Q* statistic = 6.41, *P*-heterogeneity = 0.379), and no horizontal pleiotropy was detected (Egger intercept = 0.007, *P*-intercept = 0.418) (Table S3). Leave-one-out analyze demonstrates that removing any individual SNP does not lead to significant differences in the causal effect of this MR analysis (Figure S1).

**Table 1:** MR results of the association between SGLT2 inhibition and the risk of type 2 diabetes

Outcome	Method	Number of SNPs	OR (95% CI)	*P* value
Type 2 diabetes	IVW	7	0.47 (0.33, 0.67)	3.02 × 10^−5^
	Simple mode	7	0.34 (0.16, 0.75)	0.037
	Weighted mode	7	0.51 (0.33, 0.81)	0.028
	Weighted median	7	0.48 (0.31, 0.74)	8.23 × 10^−4^

Note: SNPs = single-nucleotide polymorphisms; OR = odds ratio; CI = confidence interval.

### 3.2 Impact of SGLT2 inhibition on psychiatric disorders

**Figure 2:**
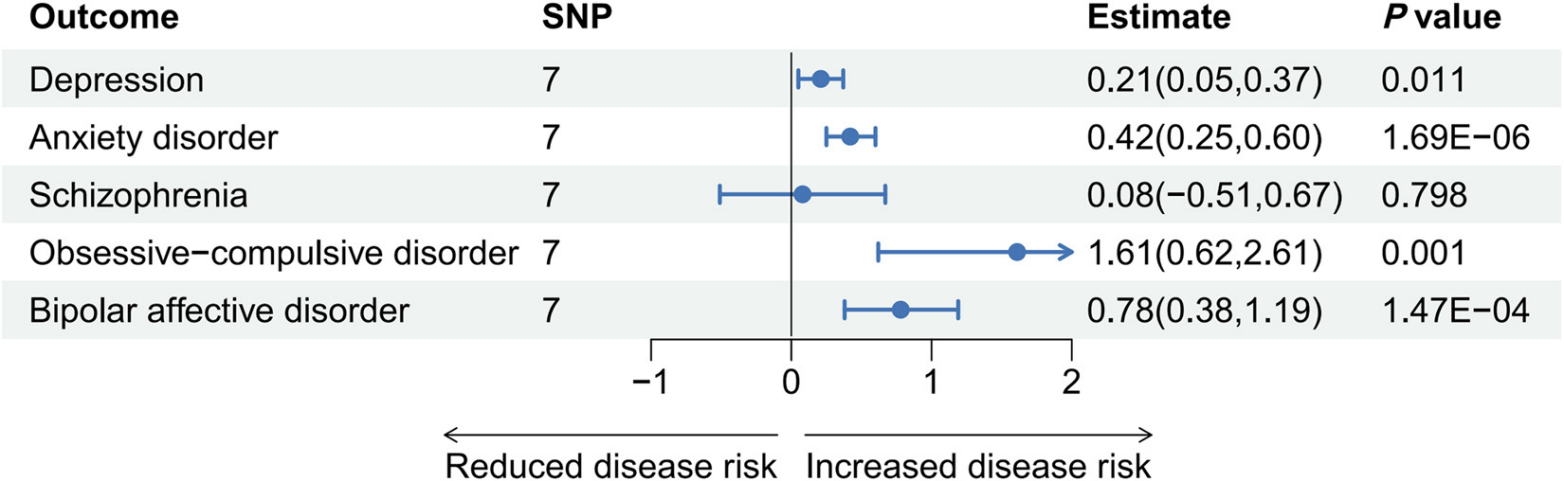
The effect of SGLT2 inhibition on psychiatric disorders. Estimate = log_10_OR.

The summary statistics for phenotypic exposures and outcomes show that the mean, standard deviations, and proportions are consistent with the expected distributions in populations of European ancestry. Through MR analysis, a significant association was identified between SGLT2 inhibition and an increased risk of anxiety disorder (*P* = 1.69 × 10^−6^, OR[95% CI] = 2.65[1.78, 3.96]), obsessive-compulsive disorder (*P* = 0.001, OR[95% CI] = 41.04[4.16, 405.27]), and bipolar affective disorder (*P* = 1.47 × 10^−4^, OR[95% CI] = 6.07[2.39, 15.39]) (Table S4 and Figure 2, considering the large differences in OR in the MR analysis results, a logarithmic transformation [estimate = log_10_OR] was applied to facilitate visualization in the figure). However, the effect of SGLT2 inhibition on depression (*P* = 0.011, OR[95% CI] = 1.63[1.12, 2.37]) did not reach Bonferroni-corrected significance levels (Table S4 and Figure 2). No association was found between SGLT2 inhibition and schizophrenia (*P* = 0.798, OR[95% CI] = 1.19[0.31, 4.66]) (Table S4 and Figure 2). Neither heterogeneity nor horizontal pleiotropy was detected in any of the above MR analyses (Table S5). Leave-one-out analysis indicated that removing any individual SNP did not significantly alter the causal effects of the positive findings in the MR analysis (Figure S1).

### 3.3 Colocalization analysis for SGLT2 inhibition

A colocalization analysis was conducted to investigate the association between SGLT2 inhibition and depression, anxiety disorder, obsessive-compulsive disorder, and bipolar affective disorder. The results indicated a posterior probability of 50.90% for SGLT2 inhibition and obsessive-compulsive disorder sharing the same causal variant (Table S6). However, no strong evidence suggesting colocalization between SGLT2 inhibition and depression (probability of sharing the same causal variant: 0.54%), anxiety disorder (probability: 6.61%), or bipolar affective disorder (probability: 5.65%) was observed (Table S6).

### 3.4 Impact of HbA1c on psychiatric disorders

**Figure 3:**
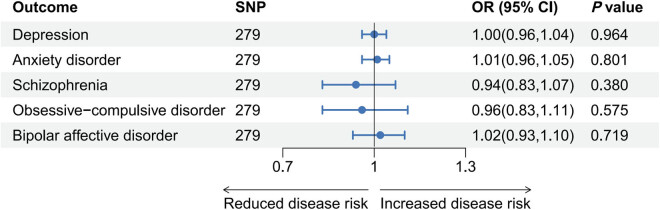
The effect of HbA1c on psychiatric disorders.

This study did not find a causal association between SGLT2 inhibition and depression, anxiety disorder, schizophrenia, obsessive-compulsive disorder, or bipolar affective disorder (Figure 3 and Table S7).

## 4 Discussion

SGLT2 is primarily found in the epithelial cells of the proximal convoluted tubule of the kidney, where it facilitates 90% of glucose reabsorption in the kidney. By inhibiting SGLT2 in the proximal convoluted tubule, SGLT2 inhibitors can suppress the reabsorption of glucose, thereby reducing blood glucose levels [26]. As the current star anti-diabetic drug, SGLT2 inhibitors have an impact on diseases of multiple systems, and their protective effects on the heart and kidney have been confirmed by numerous large-scale clinical studies [27–29]. In recent years, the impact of SGLT2 inhibitors on psychiatric disorders has received increasing attention [9,30,31]. However, there is currently no comprehensive study investigating the causal relationship between SGLT2 inhibitors and psychiatric disorders. In this study, a comprehensive analysis of the potential impact of SGLT2 inhibitors on various psychiatric disorders was conducted through MR analysis, utilizing quantitative trait loci (QTL) and GWAS summary data. Our findings suggest that SGLT2 inhibitors may increase the risk of obsessive-compulsive disorder, anxiety disorder, and bipolar affective disorder.

The results of this study indicate that SGLT2 inhibitors increase the risk of obsessive-compulsive disorder, which has been verified through colocalization analysis. Obsessive-compulsive disorder is a severe and disabling psychiatric disorder characterized by intrusive obsessions and/or compulsions that are both disturbing and time-consuming [32]. Obsessions refer to intrusive and unwanted images, thoughts, or urges that are associated with anxiety, distress, disgust, and/or a sense of something being not quite right [33]. Our research suggests that SGLT2 inhibitors may increase the risk of obsessive-compulsive disorder through some mechanism beyond their hypoglycemic effect, indicating that the use of SGLT2 inhibitors may be an important risk factor for obsessive-compulsive disorder. Common anti-anxiety drugs such as benzodiazepines are ineffective in the treatment of obsessive-compulsive disorder. Current pharmacological treatments for obsessive-compulsive disorder include selective serotonin reuptake inhibitors and tricyclic anti-depressants like clomipramine [32,34]. Discovering new therapeutic targets for obsessive-compulsive disorder treatment holds significant importance. The results of this study also indirectly suggest that SGLT2 may be a potential drug target for obsessive-compulsive disorder treatment.

Recent observational studies have shown that SGLT2 inhibitors may help reduce the risk of depression [9,10]. SGLT2 inhibitors may alleviate depression by acting on the lateral habenula and regulating serotonergic activity in the dorsal raphe nucleus [31]. However, this study showed no statistically significant difference in the impact of SGLT2 inhibitors on depression. Observational studies often suffer from biases due to the inevitable presence of confounding factors, which may affect the accuracy of research results. Additionally, patient compliance cannot always be guaranteed in observational studies of monotherapy, which can further impact the research outcomes [35]. Moreover, the aforementioned studies also have inherent information biases such as coding errors and data missingness [9]. In contrast, MR analysis can better address these issues that affect research accuracy due to the random distribution of genetic variations and lifetime exposure of genetic instruments, making the results more credible [12].

This study demonstrates that SGLT2 inhibitors increase the risk of anxiety disorder and bipolar affective disorder, but are unrelated to the risk of schizophrenia. This suggests potential side effects of long-term use of SGLT2 inhibitors. No evidence was found to correlate HbA1c with the aforementioned diseases, indicating that the effects of SGLT2 inhibitors on anxiety disorder and bipolar affective disorder are independent of their hypoglycemic action. Currently, there is a lack of high-quality research on the effects of SGLT2 inhibitors on diseases like schizophrenia and bipolar disorder [36], and this study fills an important gap in this field.

This study is the first to investigate the causal relationship between SGLT2 inhibition and five types of psychiatric disorders using MR analysis and colocalization analysis. The MR method leverages the random allocation of genetic variations to effectively reduce confounding bias from comorbid factors. QTL data and GWAS summary data were extracted, and a rigorous instrument selection and validation process was adopted, ensuring the high reliability of the research results. However, this study still has several limitations. First, the use of genetic variations as proxies for the effects of SGLT2 inhibition mainly reflects lifelong changes, which cannot be equated with the impact of short-term use of SGLT2 inhibitors [37,38]. Second, this study analyzed data derived from individuals of European ancestry. Given potential differences in genetic architecture and environmental exposures across racial/ethnic groups, the generalizability of the current findings to other populations (e.g., Asian, African ancestry) requires further validation. Future investigations should utilize large-scale cohort data from specific populations to evaluate the generalizability of these associations. Third, when considering multiple corrections, the statistical power for detecting false negatives may be reduced. Therefore, the interpretation of the results should be approached with caution [39,40]. Despite the aforementioned limitations, this study still provides a credible speculation on the causal relationship between SGLT2 inhibition and various psychiatric disorders. This study focuses on the genetic proxy effects of SGLT2 inhibition, reflecting the long-term cumulative impacts of SGLT2 inhibition on psychiatric disorders. When conducting SGLT2-related research and clinical applications, attention should be paid to the potential contribution of medication adverse effects to psychiatric symptoms. Future research should evaluate psychiatric adverse event reports from clinical trials investigating SGLT2 inhibitor use.

In conclusion, this study has revealed that SGLT2 inhibition can increase the risk of obsessive-compulsive disorder, anxiety disorder, and bipolar affective disorder through mechanisms beyond its hypoglycemic effects. The use of SGLT2 inhibitors may be a risk factor for these three disorders. These findings provide genetic evidence for the impact of SGLT2 inhibition on psychiatric disorders and serve as a basis for further mechanistic and clinical research in the future.

## Abbreviations

GTEx: genotype-tissue expression
GWAS: genome-wide association studies
HbA1c: glycated hemoglobin
IVW: inverse-variance weighted
MR: Mendelian randomization
OR: odds ratio
SGLT2: sodium–glucose cotransporter 2
SNPs: single-nucleotide polymorphisms

## Supplementary Material

Supplementary material
