# Supplementary material for: Association of SGLT2 inhibition with psychiatric disorders: A Mendelian randomization study
Source: Open Med (Wars). 2025 Oct 25;20(1):20251278. doi: 10.1515/med-2025-1278 (PMC12596861; doi:10.1515/med-2025-1278)
Supplement: Supplementary material [file med-2025-1278-sm.pdf]

Supplementary material

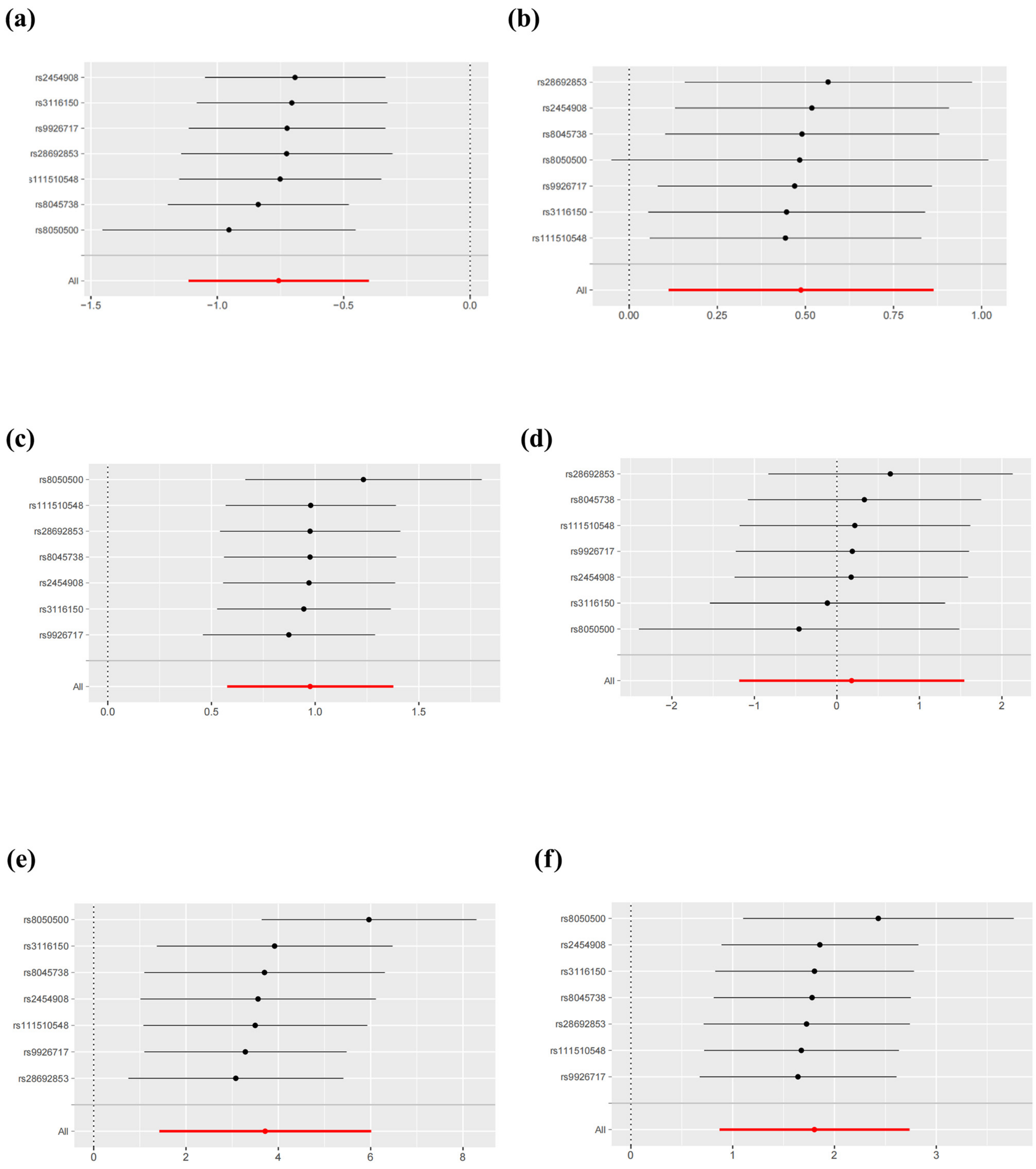

Figure S1: Leave-one-out analysis of SGLT2 inhibition on (a) Type 2 diabetes. (b) Depression (c) Anxiety disorder. (d) Schizophrenia. (e) Obsessive-compulsive disorder. (f) Bipolar affective disorder.

Table S1: Detailed information for genome-wide association study (GWAS) statistics used in the present study

Phenotype	Consortium	Sample size	Ancestry	Year	IEU OpenGWAS ID or study reference
SGLT2 inhibition	UK Biobank	344182	European	2018	ukb-d-30750_irnt
Type 2 diabetes mellitus	Finngen Study	400197	European	2023	https://storage.googleapis.com/finngen-public-data-r10/summary_stats/finngen_R10_T2D.gz
depression	Finngen Study	406986	European	2023	https://storage.googleapis.com/finngen-public-data-r10/summary_stats/finngen_R10_F5_DEPRESSIO.gz
anxiety disorder	Finngen Study	346542	European	2023	https://storage.googleapis.com/finngen-public-data-r10/summary_stats/finngen_R10_KRA_PSY_ANXIETY_EXMORE.gz
schizophrenia	Finngen Study	405094	European	2023	https://storage.googleapis.com/finngen-public-data-r10/summary_stats/finngen_R10_F5_SCHZPHR.gz
obsessive-compulsive disorder	Finngen Study	370229	European	2023	https://storage.googleapis.com/finngen-public-data-r10/summary_stats/finngen_R10_F5_OCD.gz
bipolar affective disorder	Finngen Study	366859	European	2023	https://storage.googleapis.com/finngen-public-data-r10/summary_stats/finngen_R10_F5_BIPO.gz

Table S2: Instrumental variables for SGLT2 inhibition

SNP	Beta	Se	*P* value	Effect allele frequency	maf	Effect_allele	Other_allele	Samplesize	*F*-statistics	R2-variance explained
rs111510548	0.015	0.004	6.69×10^{-5}	0.103	0.103	C	T	344182	14.757	4.29×10^{-5}
rs2454908	0.010	0.002	2.56×10^{-5}	0.565	0.435	T	C	344182	16.223	4.71×10^{-5}
rs28692853	0.015	0.002	2.78×10^{-10}	0.507	0.493	A	C	344182	36.423	1.06×10^{-4}
rs3116150	−0.014	0.003	4.16×10^{-7}	0.241	0.241	A	G	344182	23.354	6.78×10^{-5}
rs8045738	0.010	0.002	2.94×10^{-5}	0.694	0.306	G	T	344182	15.950	4.63×10^{-5}
rs8050500	0.027	0.002	1.15×10^{-30}	0.446	0.446	C	T	344182	120.677	3.50×10^{-4}
rs9926717	0.011	0.003	9.61×10^{-6}	0.284	0.284	G	A	344182	17.966	5.22×10^{-5}

Table S3: Heterogeneity and pleiotropy tests for the effect of SGLT2 inhibition on type 2 diabetes

Outcome	Method	Q statistic	*P*-heterogeneity	Egger intercept	*P*-intercept
type 2 diabetes	Inverse variance weighted	6.41	0.379		
	MR Egger	5.545	0.353	0.007	0.418

Table S4: Mendelian randomization results of the association between SGLT2 inhibition and the risk of psychiatric disorders

Outcome	Method	Number of SNPs	OR (95% CI)	*P* value
Depression	Inverse variance weighted	7	1.63 (1.12, 2.37)	0.011
	Simple mode	7	1.63 (0.85, 3.12)	0.195
	Weighted mode	7	1.60 (0.97, 2.63)	0.115
	Weighted median	7	1.62 (1.02, 2.58)	0.043
Anxiety disorder	Inverse variance weighted	7	2.65 (1.78, 3.96)	1.69×10^{-6}
	Simple mode	7	2.67 (1.19, 5.98)	0.054
	Weighted mode	7	2.07 (1.20, 3.57)	0.040
	Weighted median	7	2.43 (1.46, 4.04)	6.17×10^{-4}
Schizophrenia	Inverse variance weighted	7	1.19 (0.31, 4.66)	0.798
	Simple mode	7	1.15 (0.06, 20.43)	0.927
	Weighted mode	7	1.97 (0.31, 12.64)	0.503
	Weighted median	7	1.63 (0.32, 8.32)	0.554
Obsessive-compulsive disorder	Inverse variance weighted	7	41.04 (4.16, 405.27)	0.001
	Simple mode	7	1347.04 (5.46, 332222.70)	0.043
	Weighted mode	7	5.34 (0.60, 47.42)	0.183
	Weighted median	7	12.74 (1.42, 114.55)	0.023
Bipolar affective disorder	Inverse variance weighted	7	6.07 (2.39, 15.39)	1.47×10^{-4}
	Simple mode	7	5.59 (0.75, 41.81)	0.144
	Weighted mode	7	4.08 (1.10, 15.10)	0.079
	Weighted median	7	4.57 (1.45, 14.36)	0.009

Table S5: Heterogeneity and pleiotropy tests for the effect of SGLT2 inhibition on psychiatric disorders

Outcome	Method	*Q* statistic	*P*-heterogeneity	Egger intercept	*P*-intercept
Depression	Inverse variance weighted	2.650	0.851		
	MR Egger	2.624	0.758	0.001	0.878
Anxiety disorder	Inverse variance weighted	4.191	0.651		
	MR Egger	2.537	0.771	−0.011	0.255
Schizophrenia	Inverse variance weighted	5.009	0.543		
	MR Egger	4.438	0.488	0.022	0.484
Obsessive-compulsive disorder	Inverse variance weighted	11.791	0.067		
	MR Egger	6.196	0.288	−0.084	0.087
Bipolar affective disorder	Inverse variance weighted	4.006	0.676		
	MR Egger	3.008	0.699	−0.020	0.364

Table S6: Colocalization analysis for SGLT2 inhibition

Psychiatric disorder class	PP.H0	PP.H1	PP.H2	PP.H3	PP.H4
Depression	0.02%	83.6%	0.00%	15.8%	0.54%
Anxiety disorder	0.02%	69.8%	0.01%	23.5%	6.61%
Obsessive-compulsive disorder	0.01%	34.30%	0.00%	14.80%	50.90%
Bipolar affective disorder	0.02%	82.1%	0.00%	12.2%	5.65%

Table S7: Mendelian randomization results of the association between HbA1c and the risk of psychiatric disorders

Outcome	Method	Number of SNPs	OR (95% CI)	*P* value
Depression	Inverse variance weighted	279	1.00 (0.96, 1.04)	0.964
	Simple mode	279	0.94 (0.82, 1.07)	0.333
	Weighted mode	279	0.97 (0.91, 1.02)	0.244
	Weighted median	279	0.94 (0.89, 1.00)	0.047
Anxiety disorder	Inverse variance weighted	279	1.01 (0.96, 1.05)	0.801
	Simple mode	279	1.10 (0.95, 1.27)	0.198
	Weighted mode	279	1.04 (0.98, 1.11)	0.192
	Weighted median	279	1.05 (0.98, 1.12)	0.169
Schizophrenia	Inverse variance weighted	279	0.94 (0.83, 1.07)	0.380
	Simple mode	279	1.19 (0.71, 1.98)	0.504
	Weighted mode	279	1.07 (0.85, 1.35)	0.543
	Weighted median	279	1.14 (0.91, 1.42)	0.270
Obsessive-compulsive disorder	Inverse variance weighted	279	0.96 (0.83, 1.11)	0.575
	Simple mode	279	0.87 (0.51, 1.46)	0.589
	Weighted mode	279	0.99 (0.77, 1.28)	0.956
	Weighted median	279	1.05 (0.82, 1.34)	0.720
Bipolar affective disorder	Inverse variance weighted	279	1.02 (0.93, 1.10)	0.719
	Simple mode	279	0.95 (0.69, 1.30)	0.746
	Weighted mode	279	0.93 (0.80, 1.08)	0.329
	Weighted median	279	0.90 (0.78, 1.04)	0.157
